# Influence of Two-Stage Combinations of Constructed Wetlands on the Removal of Antibiotics, Antibiotic Resistance Genes and Nutrients from Goose Wastewater

**DOI:** 10.3390/ijerph16204030

**Published:** 2019-10-21

**Authors:** Xiaofeng Huang, Yi Luo, Zuolan Liu, Changlian Zhang, Hang Zhong, Jiajia Xue, Qigui Wang, Zhiping Zhu, Chao Wang

**Affiliations:** 1 Poultry Science Institute, Chongqing Academy of Animal Sciences, Chongqing 402460, China; hxf2007@yeah.net (X.H.); homleestar@163.com (Y.L.); liuzuolan9229@163.com (Z.L.); zcl0326@163.com (C.Z.); hangzhongcqaas@163.com (H.Z.); xuejiajia25@163.com (J.X.); wangqigui@hotmail.com (Q.W.); 2 Scientific Observation and Experiment Station of Livestock Equipment Engineering in Southwest, Ministry of Agriculture, Chongqing 402460, China; 3 Institute of Environment and Sustainable Development in Agriculture, Chinese Academy of Agricultural Sciences, Beijing 100081, China

**Keywords:** hybrid CWs, combination, antibiotics, antibiotic resistance genes, removal

## Abstract

Antibiotic and antibiotic resistance genes (ARGs) have been considered as emerging environmental contaminants and possess potential crisis to global public health. However, little is known about the differences between various configurations of two-stage combinations of constructed wetlands (CWs) on antibiotics and ARG removal from wastewater. In the study, three configurations of two-stage hybrid CWs (horizontal subsurface flow-down-flow vertical subsurface flow CWs, HF-DVF; horizontal subsurface flow-up-flow vertical subsurface flow CWs, HF-UVF; down-flow vertical subsurface flow-up-flow vertical subsurface flow CWs, DVF-UVF) were operated to evaluate their ability to remove high-concentration antibiotics (tilmicosin—TMS and doxycycline—DOC), ARGs (seven *tet* genes and three *erm* genes), *intI*1, 16S rRNA, and nutrients from goose wastewater. The results showed that all three hybrid CWs could remove more than 98% of TMS and DOC from wastewater, without significant difference among treatments (*p* > 0.05). For ARGs, DVF-UVF showed significantly higher removal efficiencies of *intI*1, *erm*B, *erm*C, *erm*F, *tet*W, and *tet*G compared to HF-UVF (*p* < 0.05), mainly because they might remove and arrest growth of bacteria. The relatively high removal efficiencies of NH_4_^+^-N, NO_3_^—^N, and NO_2_^-^-N were also observed from DVF-UVF, ranging from 87% to 95% (*p* > 0.05), indicating that anaerobic ammonium oxidation (anammox) might be established in the CWs. Our results demonstrate that the removal performances of antibiotics using two-stage hybrid CWs are not affected by the combined configuration, whereas the combination of DVF and UVF CWs perform better on the removal of ARGs and nutrients compared with HF-DVF and HF-UVF CWs.

## 1. Introduction

Antibiotics have been widely used in animal for prophylaxis, therapy, and growth promotion [1,2]. However, due to the over consumption and poor absorption, the majority of antibiotics have been continuously released into the environment and maintain permanent presence [3,4]. As a consequence, the antibiotic resistant bacteria and antibiotic resistant genes (ARGs) emerge and spread more rapidly in the environment, and threaten the ecological balance [5,6]. Therefore, the antibiotic and ARGs have been considered as emerging environmental contaminants and potential crises to global public health [7]. In recent years, veterinary antibiotics have been detected in the soil, vegetables, river, and sea, and the highest antibiotic concentration reached 47.0 mg/L in the Yangtze river of China [8,9,10]. Correspondingly, high frequency and abundance of ARGs have also been observed in the environment [11,12]. 

Constructed wetlands (CWs) have been proven as an effective system for removing of antibiotics and ARGs in wastewater, with low maintenance costs and environment-friendly features [13,14,15]. However, the composition of wastewater became complex in recent years and pollutants could not be treated well by single CW. Then, hybrid CWs that combine different types of CWs were introduced to obtain high removal efficiencies of pollutants [16,17]. Results have confirmed that hybrid CWs performed better in nitrogen removal than single CW, because of the combination of nitrification and denitrification provided by various types of CWs [16,18]. Moreover, hybrid CWs have also been proved to enhance the removal efficiencies of antibiotics and ARGs compared to single CW [13,19]. Due to the various characteristics of single CW, the different configurations of hybrid CWs could possess distinct microsystems and functions [20]. However, few researches were focused on exploring the differences among various configurations of hybrid CWs on the removal performances of antibiotics and ARGs from wastewater. Recently, horizontal subsurface flow (HF), down-flow vertical subsurface flow (DVF) and up-flow vertical subsurface flow (UVF) CWs were proved to be efficient on wastewater antibiotics and ARG removal, and they have their own advantages regarding pollutant removal [3,21]. Accordingly, we want to combine the three types of CWs (HF, UVF, and DVF) to compose different hybrid CWs (HF-UVF, HF-DVF, and UVF-DVF) and compare their performances on antibiotics and ARG removal from wastewater.

In China, the combination of tilmicosin (TMS) and doxycycline (DOC) has been widely used to cure the respiratory diseases, which is high prevalence and infectious in poultry [22]. As a result, TMS and DOC were detected in the wastewater and researches showed that CWs could efficiently remove the TMS and DOC at a low concentration from wastewater (36 ng/L for TMS and 180 ng/L for DOC) [23,24]. However, the dosage of TMS and DOC for cure in geese are increasing as time went on due to the drug resistance of bacteria. Besides, in China, the annual inventory amount of geese was more than 580 million and the daily volume of wastewater was huge owing to the habit of playing with water [25]. Therefore, it is necessary to investigate the removal efficiencies of high-concentration TMS and DOC by CWs from goose wastewater. Moreover, seven *tet* genes (*tet*O, *tet*Q, *tet*W, *tet*A, *tet*C, *tet*G, and *tet*X) and three *erm* genes (*erm*B, *erm*F, and *erm*C) were chosen as representatives of tetracycline (including TMS)—resistance and macrolide (including DOC)—resistance mechanisms due to their frequent occurrence in wastewater [26,27]. 

Hence, the main objectives of this study were: (1) to compare the removal efficiencies of high-concentration TMS and DOC and ARGs in three configurations of two-stage hybrid CWs (HF-UVF, HF-DVF, and UVF-DVF) form goose waster; and (2) to investigate the removal efficiencies of nutrients in goose wastewater by three configurations of two-stage hybrid CWs.

## 2. Materials and Methods 

### 2.1. Design of Hybrid CWs

The experiment was conducted in six CWs including two HF-CWs, two UVF-CWs and two DVF-CWs. All the CWs were set up in stainless steel containers. HF-CWs (0.9 m height × 0.43 m width × 0.7 m length) were composed of 20 cm bottom layer of gravel, 40 cm middle layer of ceramsite and zeolite (v:v = 1:1) and 20 cm top layer of red soil. vertical subsurface flow constructed wetlands (VF-CWs) (diameter 0.62 m, height 0.9 m) were filled with three layers (from lower to upper): 20 cm layer of gravel, 40 cm layer of ceramsite and zeolite (v:v = 1:1), and 20 cm layer of red soil. All CWs were planted with *Phragmites australis* on the top layers. Then, every two types of the CWs were connected into three configurations of two-stage hybrid CWs (HF-UVF, HF-DVF, and DVF-UVF).

The experiment was performed in the poultry scientific research base of Chongqing Academy of Animal Science, Chongqing City, China. Goose wastewater was obtained from sedimentation tank of the research base, having an average 25.55 mg/L total nitrogen (TN), 5.53 mg/L nitrate nitrogen (NO_3_^-^-N), 1.59 mg/L nitrite nitrogen (NO_2_^-^-N), 4.74 mg/L ammonia nitrogen (NH_4_^+^-N), 13.39 mg/L total phosphorus (TP), 25.65 mg/L chemical oxygen demand (COD), 10,461.54 ng/L TMS, and 125.54 ng/L DOC. According to the ratio of antibiotics in real wastewater and the dosage for cure in practice, the physicochemical properties of TMS and DOC were spiked into the wastewater to produce individual high concentrations of about 2,500,000 and 30,000 ng/L. The synthetic wastewater was prepared at day 0 and day 42 and stocked in a cistern, and then continuously infused into each hybrid CW by peristaltic pump at a flow rate of 6.25 mL/min. The corresponding theoretical hydraulic residence time (HRT) was 6 days in each hybrid CWs (3 days by each single CW). This experiment was initiated in August 2018 and lasted for 84 days. In this period, samplings were carried out on day 21, 42, 63, and 84 at 8:00, 12:00, and 16:00, collecting the influent and effluent in each hybrid CWs. 

### 2.2. Analysis of TMS and DOC

The antibiotics in wastewater were analyzed following the methods described by Huang et al. [28]. Water samples were filtered through 0.45 µm mixed cellulose ester filters and adjusted to pH 4.0 by HCL, after which 0.2 g/L Na_2_EDTA was added. Then, samples were extracted using 200mg/6mL Oasis HLB extraction cartridges, and then washed by deionized water and dried under a gentle stream air. The dried samples were eluted by pure methanol, dried under a gentle stream of nitrogen gas at 40 °C and re-dissolved in 1mL of 20% methanol for subsequent LC-MS/MS analysis. A reverse phase C-18 column (Kinetex, 2.6µm, 100 × 4.6 mm, Phenomenex, USA) was used to separate the target antibiotics (TMS and DOC). A binary gradient consisted of 0.5% formic acid water solution for mobile phase A and pure methanol for mobile phase B. Electrospray Ionization (ESI) positive complete scan mode was used for detecting the antibiotics. The quantification of antibiotics was determined using external standard calibration curves and the correlation coefficients were more than 0.99. 

### 2.3. Analysis of 16S rRNA, IntI1, and ARGs

The DNA in wastewater were extracted following the methods described by Sui et al. [29]. Water samples were passed through 0.22µm filter membrane, and then extracted total DNA using FastDNA SPIN Kit for Soil (MP Biochemicals, Solon, OH, USA) according to the manufacturer’s instructions. NanoDrop ND-1000 spectrophotometer (NanoDrop Technologies, Willmington, DE, USA) and 2% agarose gel electrophoresis were used to determine the concentration and qualify of DNA products. Seven *tet* genes (*tet*O, *tet*Q, *tet*W, *tet*A, *tet*C, *tet*G, and *tet*X), three *erm* genes (*erm*B, *erm*F, and *erm*C), integrase gene of Class 1 integrons (*intI*1) and 16S rRNA gene were quantified using SYBR Green qPCR mix (Takara, Ostu, Japan) and ABI 7500 Real-time PCR system (Applied Biosystems, Foster City, CA, USA). Primer sequences and annealing temperatures targeting these genes are provided in Table S1. The real-time PCR program was as follows: 30 s at 95°C, followed by 40 cycles of 5 s at 95°C, 34 s at the annealing temperatures, and 15 s at 95°C. Plasmids carrying these selected genes were used to make calibration curves and the correlation coefficients were higher than 0.99. Melting curve analysis and gel electrophoresis were used to confirm product specificity. All PCR reactions were carried out in triplicate.

### 2.4. Analysis of Nutrients

Water nutrients including TN, NO_3_^-^-N, NO_2_^-^-N, NH_4_^+^-N, COD, and TP were determined according to the standard methods for the examination of water and wastewater [30].

### 2.5. Calculation of Removal Efficiencies and Statistical Analysis

The mass removal efficiency (%) of antibiotics, ARGs, and nutrients were calculated according to the formula

Removal efficiency (%) = ((Mi − Me) / Mi) × 100,(1)

where Mi and Me represent the concentration of antibiotics, ARGs, and nutrients in the influent and effluent respectively.

All experimental values were expressed as means of three measurements with standard deviation. Analysis of variance (ANOVA) in Software SPSS 22.0 (SPSS Inc., Chicago, IL, USA) was used for all statistical analyses, including Bartlett’s test for homogeneity of variances analysis and Duncan’s test for differences between means. All statements of differences were based on a significance level of *p* < 0.05.

## 3. Results

### 3.1. Removal Efficiencies of TMS and DOC in Hybrid CWs

**Figure 1:**
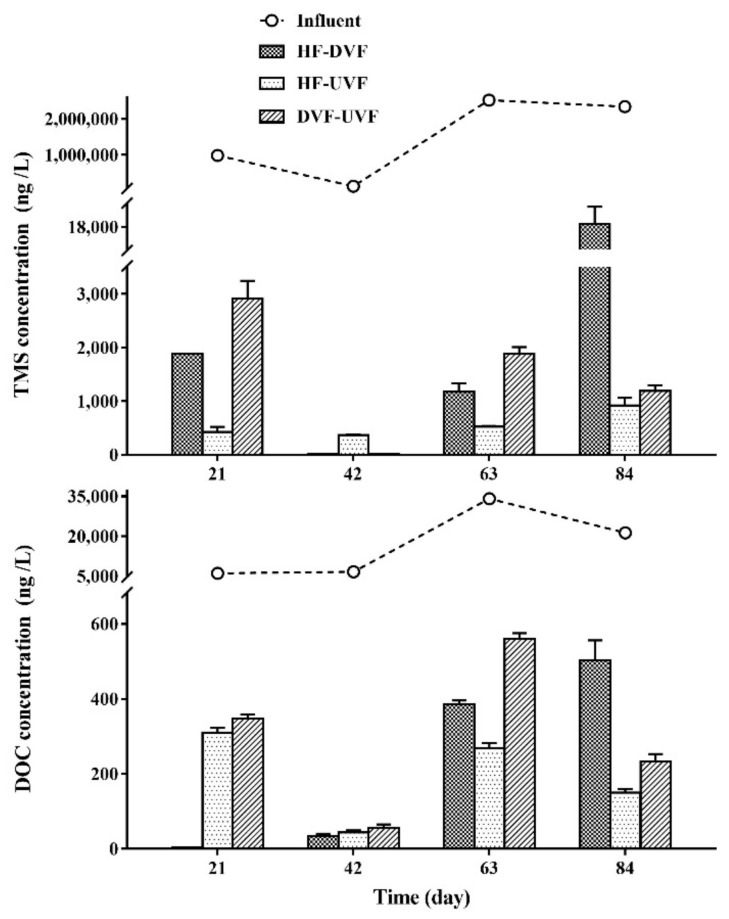
TMS and DOC concentrations in the influent and effluent of different hybrid constructed wetlands. HF-DVF: horizontal subsurface flow-down-flow vertical subsurface flow CWs; HF-UVF: horizontal subsurface flow-up-flow vertical subsurface flow CWs; DVF-UVF: down-flow vertical subsurface flow-up-flow vertical subsurface flow CWs.

The TMS and DOC concentrations in the influent and effluent of three hybrid CWs have been shown in Figure 1. In the influents, TMS was the predominant compound with the average concentration of 1,495,658.33 ng/L, while DOC was present at relatively low level, with the average concentration at 17,019.17 ng/L. The average effluent antibiotic concentrations for three hybrid CWs (HF-DVF, HF-UVF, and DVF-UVF) were 5360.88, 560.42, and 1497.32 ng/L for TMS; and 230.53, 193.08, and 298.7 ng/L for DOC, respectively. The removal efficiencies of TMS, DOC, and total antibiotics (the sum of TMS and DOC—∑antibiotics) were more than 98% for all three hybrid CWs and relatively stable along with time, and TMS removal efficiencies in three hybrid CWs were slightly better than DOC (Table 1). However, there were no significant differences among three hybrid CWs for TMS, DOC, and total antibiotics removal (*p* > 0.05), while HF-DVF presented slightly high removal efficiency for DOC.

### 3.2. Removal Efficiencies of 16S rRNA, IntI1, and ARGs in Hybrid CWs

**Figure 2:**
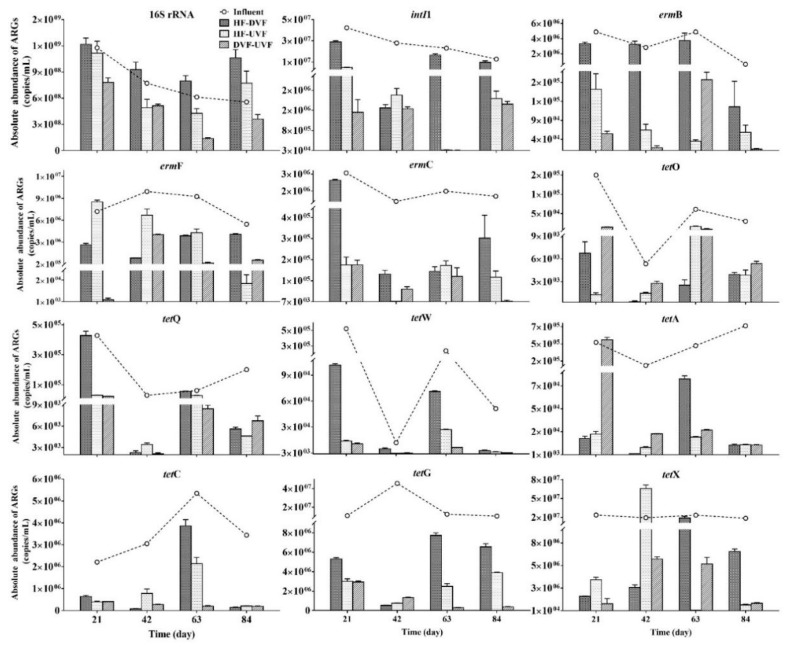
16S rRNA, *intI*1, and antibiotic resistant genes abundances in the influent and effluent of different hybrid constructed wetlands.

The 16S rRNA, seven *tet* genes (*tet*O, *tet*Q, *tet*W, *tet*A, *tet*C, *tet*G, and *tet*X), three *erm* genes (*erm*B, *erm*F, and *erm*C) and *intI*1 were detected in influents and effluents of three hybrid CWs (Figure 2). Among the selected genes, *intI*1 was found to be the most abundant gene in influents (2.65 × 10^7^ copies/mL), followed by *tet*X and *tet*G (2.12 × 10^7^ and 1.99 × 10^7^ copies/mL), while *tet*O displayed the lowest abundance (6.17 × 10^4^ copies/mL). The absolute abundances of influent 16S rRNA and *intI*1 decreased along with time, while total *tet* and *erm* genes abundances presented high variability throughout the study. 

During CWs treatment process, the removal efficiencies of 16S rRNA and total *erm* genes (∑*tet*) in DVF-UVF were significantly higher than those in HF-UVF (*p* < 0.05) (Table 1). The abundances of *intI*1, *erm*B, and *erm*C in HF-DVF and DVF-UVF were reduced by more than 90%, which were significantly higher than those in HF-UVF (*p* < 0.05). There was no significant difference for the removal efficiency of *erm*F among three hybrid CWs (*p* > 0.05).

For seven *tet* genes in three hybrid CWs, except for *tet*X in HF-UVF, the average removal efficiencies were more than 50% (Table 1). Compared to HF-UVF, DVF-UVF showed significantly higher removal efficiencies of *tet*W and *tet*G (*p* < 0.05). There were no significant differences for removal efficiencies of five *tet* genes (*tet*O, *tet*Q, *tet*A, *tet*C, and *tet*X) and total *tet* genes (∑*tet*) among three hybrid CWs (*p* > 0.05). However, DVF-UVF presented better performances in ∑*tet*, *tet*Q, *tet*A, *tet*C, and *tet*X removal than other two hybrid CWs, while the highest removal efficiencies for *tet*O and *tet*A were found in HF-UVF and HF-DVF, respectively. The removal efficiencies for all ARGs by three hybrid CWs were in the following order: DVF-UVF > HF-DVF > HF-UVF.

### 3.3. Removal Efficiencies of Nutrients in Hybrid CWs 

**Figure 3:**
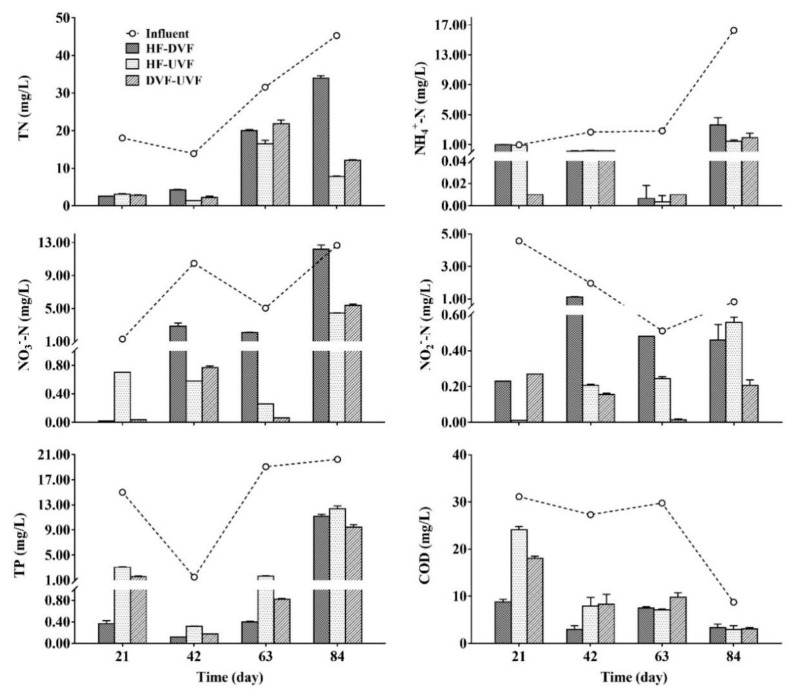
Nutrients concentrations in the influent and effluent of different hybrid constructed wetlands.

The nutrients concentrations in the influent and effluent of three hybrid CWs have been shown in Figure 3. During an operation period of 84 days, the nutrients concentrations presented high variability and the average influent concentrations of TN, NH_4_^+^-N, NO_3_^-^-N, NO_2_^-^-N, TP, and COD were 27.20, 5.69, 7.37, 1.97, 13.96, and 24.24 mg/L respectively. In effluents, TN, NH_4_^+^-N, NO_3_^-^-N, and NO_2_^-^-N were detected at the concentration of 1.03–33.99, 0.00–3.60, 0.02–12.17, and 0.01–0.56 mg/L, respectively. All three hybrid CWs showed quite good removal effects on nutrients, with the average removal efficiencies of 54–76% for TN, 68–95% for NH_4_^+^-N, 59–86% for NO_3_^-^-N, and 47–90% for NO_2_^-^-N (Table 1). The HF-UVF presented relatively higher average TN removal efficiency compared with other two CWs (*p* > 0.05), while DVF-UVF had the highest average removal efficiencies of NH_4_^+^-N, NO_3_^-^-N, and NO_2_^-^-N (95%, 87%, and 90%, respectively) among three hybrid CWs. 

In three effluents, the concentrations of COD and TP ranged from 3.01 to 31.13 and 0.12 to 11.19 mg/L, respectively (Figure 3). The COD and TP removal efficiencies in HF-DVF were 74% and 83%, which were both higher than those in HF-UVF (59% for COD and 72% for TP) and DVF-UVF (61% for COD and 82% for TP) (*p* > 0.05) (Table 1). The removal efficiencies for all nutrients by three hybrid CWs were in the following order: DVF-UVF > HF-UVF > HF-DVF. 

## 4. Discussion

In the present study, the removal efficiencies of TMS, DOC, and total antibiotics were relatively stable and higher than 98% for all three hybrid CWs. However, previous researches showed that TMS and DOC removal efficiencies were only 51% and 71% in HF CW [23,24]. These two different results indicated that compared with single CW, two-stage hybrid CWs present better performances in removing TMS and DOC from wastewater, even when the antibiotic concentrations are much higher than those in previous researches. The better performances of hybrid CWs were mainly attributed to long HRT, which would consequently promote the adsorption and degradation of antibiotics [3,31,32]. In three hybrid CWs, TMS removal efficiencies were slightly better than DOC, even TMS concentration was much higher in wastewater. Previous studies showed that antibiotics with higher log *Kow* were easily to be adsorbed by sediments and absorbed by plants [33,34,35]. Therefore, the high log *Kow* of TMS may be responsible for the relatively high removal efficiencies in the CWs. In the present study, the removal efficiencies of TMS, DOC, and total antibiotics had no significant differences among three hybrid CWs. The results indicated that the configuration of hybrid CWs had no obvious effect on antibiotics removal in wastewater, which might be due to the fact that the concentration of influent antibiotics was enough high to conceal the differences on effluent antibiotics levels among three hybrid CWs. 

*IntI*1 was common genetic assembly platform and associated with the proliferation [36]. In the present study, *intI*1 was found to be the most abundant gene, implying that *intI*1 might capture the *tet* and *erm* genes and contributed to the spread of ARGs [37]. A greater abundance of *tet*X and *tet*G were also found in the influent. Compared with other ARGs, *tet* genes had higher transition probabilities from fecal microorganisms to environmental bacteria to achieve high abundances [38]. Moreover, the proliferation of *tet*G could also be promoted by the abundant *intI*1 in wastewater [27]. As we know, the abundance of 16S rRNA could reflect the bacteria level in wastewater [39]. In the present study, DVF-UVF presented lower effluent 16S rRNA abundance and higher removal efficiency compared with HF-DVF and HF-UVF, and the differences increased as time went on. Therefore, the bacteria might be arrested growth and removed in DVF-UVF, probably because of the predominant anaerobic conditions in the DVF-UVF CWs [40]. By contrast, HF and DVF had better oxygen supply than UVF, which might promote the growth of bacteria. As a result, a higher abundance of effluent 16S rRNA was found in HF-DVF during the whole experiment period. 

Consistent with the observation of 16S rRNA, *intI*1, ∑*erm*, and ∑*tet* showed better removal efficiencies in DVF-UVF, which were both more than 87%. However, when using single UVF, the removal efficiencies for *intI*1 and *tet* genes were only around 62% [28]. The differences might also be attributed to the long HRT in hybrid CWs compared with single CW. Besides, the ARG abundances were positively correlated with antibiotic concentrations, therefore, the massive removal of TMS and DOC by hybrid CWs in the present study also contributed to the high removal efficiencies of ARGs [41,42]. Moreover, previous report attributed significant reduction in abundances of ARGs to the anaerobic environment in CWs [43]. Therefore, we speculated that the predominant anaerobic conditions in DVF-UVF CWs arrested bacteria growth and promoted the removal of *intI*1, ∑*erm*, and ∑*tet* consequently. For *erm*B, *erm*C, and *tet*W, HF-UVF and DVF-UVF showed significant higher removal efficiencies than HF-DVF. Those results also confirmed that the UVF had low oxygen supply and formed anaerobic conditions to promote the removal of the ARGs [3]. As mentioned above, we consider that compared with other two hybrid CWs, DVF-UVF performed better in ARG removal from goose wastewater. 

Previous studies indicated that HF-DVF could form aerobic-anaerobic condition and combine aerobic with anaerobic microbial degradation to benefit TP removal [20,44]. In this study, HF-DVF showed relatively higher TP and COD removal efficiencies compared with the other two hybrid CWs. The major reason might be that HF-DVF improved the degradation activity and proliferation of microbes, which was consistent with high 16S rRNA abundance in HF-DVF [45]. On the contrary, DVF-UVF had the highest removal efficiencies of NH_4_^+^-N, NO_3_^-^-N, and NO_2_^-^-N among three hybrid CWs, which were 95%, 86%, and 80% respectively. Due to the predominant anoxic-anaerobic conditions, DVF-UVF could promote the establish of anaerobic ammonium oxidation (anammox), which was an effective NH_4_^+^-N and NO_2_^-^-N removal pathway [40,46]. Previous research also verified that the major N was removed by anammox process in DVF-UVF CWs [47]. Therefore, we believe that anammox might be established in DVF-UVF and contributed to the removal of NH_4_^+^-N, NO_3_^—^N, and NO_2_^-^-N. Besides, the results also indicate that organic N might not be effectively removed by DVF-UVF because of TN removal efficiencies was relatively low and it might also contribute to the high COD in DVF-UVF.

## 5. Conclusions

Our results clarified the effect of two-stage combinations of CWs on removal of high-concentration antibiotics, ARGs, and nutrients from goose wastewater. The main conclusions are listed as below:

(1) All three hybrid CWs were efficient for removing high-concentration TMS and DOC from wastewater and the combined configuration of hybrid CWs has no significant effect on the removal of antibiotics;
(2) DVF-UVF CWs possessed better ARG removal efficiencies from wastewater, probably due to the inhibition of bacterial growth;
(3) DVF-UVF CWs might promote the establish of anammox and benefit for removing N from wastewater;
(4) DVF-UVF CWs was the optimal choice for removing pollutants from goose wastewater.

## Figures and Tables

**Table 1:** Average removal efficiencies of the studied antibiotics, antibiotic resistance genes, and nutrients obtained in each hybrid constructed wetland ^1^

Items ^2^	HF-DVF (%)	HF-UVF (%)	DVF-UVF (%)
Antibiotics			
TMS	100 ± 0	100 ± 0	100 ± 0
DOC	99 ± 1	98 ± 2	98 ± 2
∑antibiotics	100 ± 0	100 ± 0	100 ± 0
Genes			
16S rRNA	−36 ± 38 ^b^	8 ± 34 ^ab^	45 ± 22 ^a^
*intI*1	44 ± 33 ^b^	91 ± 7 ^a^	94 ± 6 ^a^
*erm*B	30 ± 39 ^b^	96 ± 4 ^a^	98 ± 2 ^a^
*erm*F	58 ± 29	42 ± 49	85 ± 19
*erm*C	64 ± 30 ^b^	92 ± 5^a^	93 ± 4 ^a^
∑*erm*	49 ± 13 ^b^	64 ± 25 ^ab^	88 ± 14 ^a^
*tet*O	93 ± 4	83 ± 13	76 ± 19
*tet*Q	50 ± 52	83 ± 20	93 ± 5
*tet*W	68 ± 18 ^b^	89 ± 7 ^a^	91 ± 8 ^a^
*tet*A	94 ± 8	96 ± 2	67 ± 51
*tet*C	73 ± 32	78 ± 14	91 ± 7
*tet*G	57 ± 29^b^	79 ± 15 ^ab^	91 ± 12 ^a^
*tet*X	66 ± 33	10 ± 168	85 ± 13
∑*tet*	65 ± 29	64 ± 42	90 ± 4
Nutrients			
TN	54 ± 28	76 ± 19	68 ± 25
NH_4_^+^-N	68 ± 46	73 ± 44	95 ± 5
NO_3_^-^-N	59 ± 40	75 ± 24	86 ± 20
NO_2_^-^-N	47 ± 37	69 ± 31	90 ± 10
TP	83 ± 26	72 ± 23	82 ± 19
COD	74 ± 11	59 ± 25	61 ± 13

^1^ Data are shown as mean ± SD and each mean represents 12 samples (3 samples at each time point). ^2^ HF-DVF, horizontal subsurface flow-down-flow vertical subsurface flow CWs; HF-UVF, horizontal subsurface flow-up-flow vertical subsurface flow CWs; DVF-UVF, down-flow vertical subsurface flow-up-flow vertical subsurface flow CWs; TMS, tilmicosin; DOC, doxycycline; ∑antibiotics, the sum of TMS and DOC; ∑*erm*, total *erm* genes; ∑*tet*, total *tet* genes; TN, total nitrogen; NH_4_^+^-N, ammonia nitrogen; NO_3_^-^-N, nitrate nitrogen; NO_2_^-^-N, nitrite nitrogen; TP, total phosphorus; COD, chemical oxygen demand. ^a, b^ Different letters in the same row indicate significant differences among three treatments (*p* < 0.05).

## Supplementary Materials

The following are available online at https://www.mdpi.com/1660-4601/16/20/4030/s1, Table S1: Synthetic of oligonucleotides used in this study.
