# Supplementary material for: Influence of Two-Stage Combinations of Constructed Wetlands on the Removal of Antibiotics, Antibiotic Resistance Genes and Nutrients from Goose Wastewater
Source: Int J Environ Res Public Health. 2019 Oct 21;16(20):4030. doi: 10.3390/ijerph16204030 (PMC6843979; doi:10.3390/ijerph16204030)
Supplement: Supplementary file 1 [file ijerph-16-04030-s001.pdf]

International Journal of
Environmental Research and Public Health

MDPI

Table S1. Synthetic of oligonucleotides used in this study.

Target genes	Primer	5′-3′ sequence	Amplicon size / bp	Annealing temp (℃)	Reference
16S rDNA	1369-F	5′- CGGTGAATACGTTCYCGG-3'	172	53	[1]
	1541-R	5'-AAGGAGGTGATCCRGCCGCA-3'			
*intI*1	*intI*1-F	5′-CCTCCCGCACGATGATC-3′	280	55	[2]
	*intI*1-R	5′-TCCACGCATCGTCAGGC-3′			
tet O	*tet*O-F	5'-ACGGARAGTTTATTGTATACC-3'	171	45	[3]
	*tet*O-R	5'-TGGCGTATCTATAATGTTGAC-3'			
tet Q	*tet*Q-F	AGAATCTGCTGTTTGCCAGTG	167	60	[4]
	*tet*Q-R	CGGAGTGTCAATGATATTGCA			
tet W	*tet*W-F	5'-GAGAGCCTGCTATATGCCAGC-3'	168	60	[3]
	*tet*W-R	5'-GGGCGTATCCACAATGTTAAC-3'			
tet A	*tet*A-F	5'-GCTACATCCTGCTTGCCTTC-3'	210	52	[5]
	*tet*A-R	5'-CATAGATCGCCGTGA AGAGG-3'			
*tet*C	*tet*C-F	TGCAACTCGTAGGACAGGTG	139	60	[4]
	*tet*C-R	ACCAGTGACGAAGGCTTGAG			
*tet*G	*tet*G-F	CTCGGTGGTATCTCTGCTCA	147	58	[4]
	*tet*G-R	CAGAACGAATGGTTTGATGC			
tet X	*tet*X-F	5'-AGCCTTACCAATGGGTGTAAA-3'	278	52	[6]
	*tet*X-R	5'-TTCTTACCTTGGACATCCCG-3'			
*erm*B	*erm*B-F	5'-GATACCGTTTACGAAATTGG-3'	362	58	[7]
	*erm*B-R	5'-GAATCGAGACTTGAGTGTGC-3'			
*erm*C	*erm*C-F	5'-TCAAAACATAATATAGATAAA-3'	292	56	[4]
	*erm*C-R	5'-GCTAATATTGTTTAAATCGTCAAT-3'			
*erm*F	*erm*F-F	5'-CGACACAGCTTTGGTTGAAC-3'	309	56	[7]
	*erm*F-R	5'-GGACCTACCTCATAGACAAG-3'			

References

1. He, J.Z.; Shen, J.P.; Zhang, L.M.; Zhu, Y.G.; Zheng, Y.M.; Xu, M.G.; Di, H. Quantitative analyses of the abundance and composition of ammonia-oxidizing bacteria and ammonia-oxidizing archaea of a Chinese upland red soil under long-term fertilization practices. *Environ. Microbiol.* **2007**, 9, 2364-2374.
2. Goldstein, C.; Lee, M.D.; Sanchez, S.; Hudson, C.; Phillips, B.; Register, B.; Grady, M.; Liebert, C.; Summers, A.O.; White, D.G., et al. Incidence of class 1 and 2 integrases in clinical and commensal bacteria from livestock, companion animals, and exotics. *Antimicrob. Agents Chemother* **2001**, 45, 723-726, doi:10.1128/AAC.45.3.723-726.2001.
3. Aminov, R.; Garrigues-Jeanjean, N.; Mackie, R.I. Molecular ecology of tetracycline resistance: development and validation of primers for detection of tetracycline resistance genes encoding ribosomal protection proteins. *Appl. Environ. Microbiol.* **2001**, 67, 22-32.

4. Wang, N.; Guo, X.; Yan, Z.; Wang, W.; Chen, B.; Ge, F.; Ye, B. A comprehensive analysis on spread and distribution characteristic of antibiotic resistance genes in livestock farms of southeastern China. *Plos One 2016*, 11, e0156889.
5. Ng, L.-K.; Martin, I.; Alfa, M.; Mulvey, M. Multiplex PCR for the detection of tetracycline resistant genes. *Mol. Cell. Probe.* **2001**, 15, 209-215.
6. Ghosh, S.; Ramsden, S.J.; LaPara, T.M. The role of anaerobic digestion in controlling the release of tetracycline resistance genes and class 1 integrons from municipal wastewater treatment plants. *Appl. Microbiol. Biot.* **2009**, 84, 791-796.
7. Wei, B.; Huang, F.; Li, H.; Su, J. Persistence of sulfonamide and macrolide resistance genes during sewage sludge composting. *Chinese journal of apply environmental microbiology* **2014**, 20, 395-400.
